# Hierarchical spatiotemporal modeling of human visceral leishmaniasis in Rio Grande do Norte, Brazil

**DOI:** 10.1371/journal.pntd.0011206

**Published:** 2023-04-03

**Authors:** Helin G. Hernandez, Grant D. Brown, Iraci D. Lima, José F. Coutinho, Mary E. Wilson, Eliana L. T. Nascimento, Selma M. B. Jeronimo, Christine A. Petersen, Jacob J. Oleson

**Affiliations:** 1 Department of Biostatistics, University of Iowa, Iowa City, Iowa, United States of America; 2 State Health Secretariat, State Government of Rio Grande do Norte, Natal, Rio Grande do Norte, Brazil; 3 Institute of Tropical Medicine of Rio Grande do Norte, Federal University of Rio Grande do Norte, Natal, Rio Grande do Norte, Brazil; 4 Departments of Internal Medicine and Microbiology & Immunology, University of Iowa, Iowa City, Iowa, United States of America; 5 Iowa City VA Medical Center, Iowa City, Iowa, United States of America; 6 Department of Infectious Diseases, Federal University of Rio Grande do Norte, Natal, Brazil; 7 Department of Biochemistry, Federal University of Rio Grande do Norte, Natal, Brazil; 8 National Institute of Sciences and Technology of Tropical Disease, Natal, Rio Grande do Norte, Brazil; 9 Department of Epidemiology, University of Iowa, Iowa City, Iowa, United States of America; Fundacao Oswaldo Cruz, BRAZIL

## Abstract

Visceral leishmaniasis (VL) is a neglected tropical disease that is globally distributed and has the potential to cause very serious illness. Prior literature highlights the emergence and spread of VL is influenced by multiple factors, such as socioeconomic status, sanitation levels or animal and human reservoirs. The study aimed to retrospectively investigate the presence and infectiousness of VL in Rio Grande do Norte (RN), Brazil between 2007 and 2020. We applied a hierarchical Bayesian approach to estimate municipality-specific relative risk of VL across space and time. The results show evidence that lower socioeconomic status is connected to higher municipality-specific VL risk. Overall, estimates reveal spatially heterogeneous VL risks in RN, with a high probability that VL risk for municipalities within the West Potiguar mesoregion are more than double the expected VL risk. Additionally, given the data available, results indicate there is a high probability of increasing VL risk in the municipalities of Natal, Patu and Pau dos Ferros. These findings demonstrate opportunities for municipality-specific public health policy interventions and warrant future research on identifying epidemiological drivers in at-risk regions.

## Author summary

As a neglected tropical disease (NTD), Leishmaniasis contributes to poor health outcomes, suffering, and death of vulnerable populations. Leishmania infantum or Leishmania donovani is transmitted from animals to humans through the sandfly vector and can be fatal in 5% of the cases even with treatment. Visceral leishmaniasis is a severe form of Leishmaniasis. The purpose of this study was to estimate regions of high Visceral leishmaniasis risk in Rio Grande do Norte, Brazil between 2007 and 2020 to help inform public health initiatives and potential health policy interventions for this disease. Visceral leishmaniasis is a reportable disease and therefore VL cases are documented to the Brazilian Ministry of Health database through passive surveillance. Passive surveillance data can suffer from missing information; individuals may be asymptomatic, or may not seek medical care allowing for diagnosis. When modeling disease risk from passive surveillance data, assuming there is an absence of disease in regions without documented cases can underestimate disease risk. In this paper we estimate disease risk and identify areas with high-risk and increasing infection trends over time while considering data characteristics such as missing information and high correlation of risk factors.

## Introduction

Human visceral leishmaniasis (VL) is endemic in Brazil [1–12] and is recognized as a neglected tropical disease by the World Health Organization [13]. In Brazil, VL etiology stems from the protozoan *Leishmania infantum*, where the domestic dog serves as a sentinel host with the phlebotomine sand fly as the typical vector [14]. In the northeast region of Brazil, VL has historically been viewed as endemic in rural regions [15]. In recent decades the geographical epidemiology of VL has diversified with increasing VL cases occurring in more urban and peri-urban regions due to urbanization across Brazil [16].

The dramatic increase in VL incidence across Northeastern Brazil has led to more research focus on this region. Costa et al. [17] and Jeronimo et al. [18] highlight VL spread to urban areas within the northeastern region of Brazil in the last two decades of the 20^*th*^ century. Silva et al. [6] reported increasing VL cases from 2012 to 2017 in major cities of the Paraíba state in Brazil, Almeida et al. [19] observed spatial expansion of VL from 2007 to 2017 in Fortaleza, a coastal city within the Ceará state in Brazil, and da Silva Lima et al. [20] noted increasing VL fatality in the last decade in Piauí, Brazil. The state of Alagoas, Brazil also experienced geographic spread of VL from 2007 to 2018, primarily in rural areas [7]. Such trends warrant government awareness and require results from improved statistical modeling to better understand, and inform, the dynamics of VL transmission patterns to guide public health policy.

Several studies have investigated the risk factors for VL susceptibility in Northeastern Brazil by considering socioeconomic, environmental, infrastructure, and demographic information. With the strong connection between social class and VL incidence [21, 22] a common source of covariate information to model socioeconomic constructs in previous work [2, 3, 5] has been a Social Vulnerability Index [23] or the Human Development Index [24]. Vulnerability indices are a blend of several quantitative variables measuring similar constructs. Social vulnerability indices from Costa et al. [23] subjectively weigh multiple Census covariates to construct three vulnerability indices measuring infrastructure, human capital and income. In contrast, the Human Development Index created by the United Nations Development Programme [24], is a univariate index of life expectancy, education, and per capita income. While vulnerability indices may facilitate interpretability for individual constructs, the correlation between the variables that make up these indices is not mitigated. Using correlated variables as covariates in a statistical model, as seen in [2, 3, 5], can lead to issues of multicollinearity, like a loss of precision of parameter estimates which can affect hypothesis test results and model predictions, from which conclusions would be unreliable.

Studies like [1] that do not use vulnerability indices and instead model the effects of correlated variables on the outcome of interest should consider potential issues of multicollinearity. In their study, Cota et al. [1] use generalized additive mixed models (GAMM) to verify the univariate association between exploratory variables and the outcome of interest. They conduct multiple hypothesis tests to identify correlated explanatory variables that can contribute to issues of multicollinearity. When bivariate associations between exploratory variables exceeded 85%, Cota et al. [1] keep the variable with the lowest p-value from the univariate GAMM models. Aside from the inherent presence of ecological fallacy in ecological studies, we note that it may be difficult to control for all correlations between explanatory variables in the statistical model. In this study we aim to highlight a method through which correlation of explanatory variables can be mitigated. In the present study, we address issues of multicollinearity through a factor analysis which results in latent variables for which correlation is mitigated.

Additionally, previous studies [3, 4, 8] analyze incidence of VL across space, using Moran’s I, and over time via segmented or joinpoint regression. Exploratory spatial techniques like Moran’s I can help quantify the presence of spatial correlation. However, unlike regression approaches, these exploratory techniques do not explain or help to predict how the outcome of interest varies between regions based on observed risk factors. Techniques like segmented or joinpoint regression can help explain changes in the outcome based on observed risk characteristics. Still, temporal regression techniques fit separately from spatial models ignore important correlation information between and within regions that can better inform model parameter estimates and predictions. Therefore, in this study, we jointly model spatial and temporal correlations.

Furthermore, utilizing these statistical methods within a classical framework in the presence of missing information, can underestimate risk and produce incorrect spatio-temporal correlation as they require unrealistic assumptions that missing information imply absence of disease. In surveillance data, areas with no documentation of disease could indicate disease absence in that region. This phenomena could also arise from a lack of reporting, especially if cases are present in surrounding areas or are documented later in time. This issue may be addressed within the classical modeling framework were we first could predict missing data via an imputation model and then fit the desired model for inference. However, in this two-step approach the spatial dependence observed and fitted in the desired model would not be used to inform the missing data imputation. Therefore, our modeling approach within a Bayesian framework allows us to jointly model the spatial dependence in the data and address the missing characteristics in the data. The advantage of jointly modeling these two components is that we use spatial information when impute missing values via same model that is used for statistical inference.

The purpose of this manuscript is to evaluate spatio-temporal risk of VL cases between 2007 and 2020 in RN. The specific contributions are two-fold: 1) addressing issues of multicollinearity between observed Census data through Exploratory Factor Analysis (EFA) and 2) jointly modeling observed incidence of VL and imputing missing data. The second contribution is addressed by fitting our model within a Bayesian framework through which imputation efforts can be informed by spatial and temporal information.

## Materials and methods

### Ethics statement

The study was approved by the Ethical Review Board of the Federal University of Rio Grande do Norte and by CONEP (CAAE 83319618.5.0000.5537).

### Study area

The study area is a Northeastern state of Brazil named Rio Grande do Norte (RN). This region is centrally located at 14° S latitude and 51° W longitude with a total area of about 52, 796 km^2^. Rio Grande do Norte is home to approximately 3.5 million individuals across 167 municipalities.

**Fig 1:**
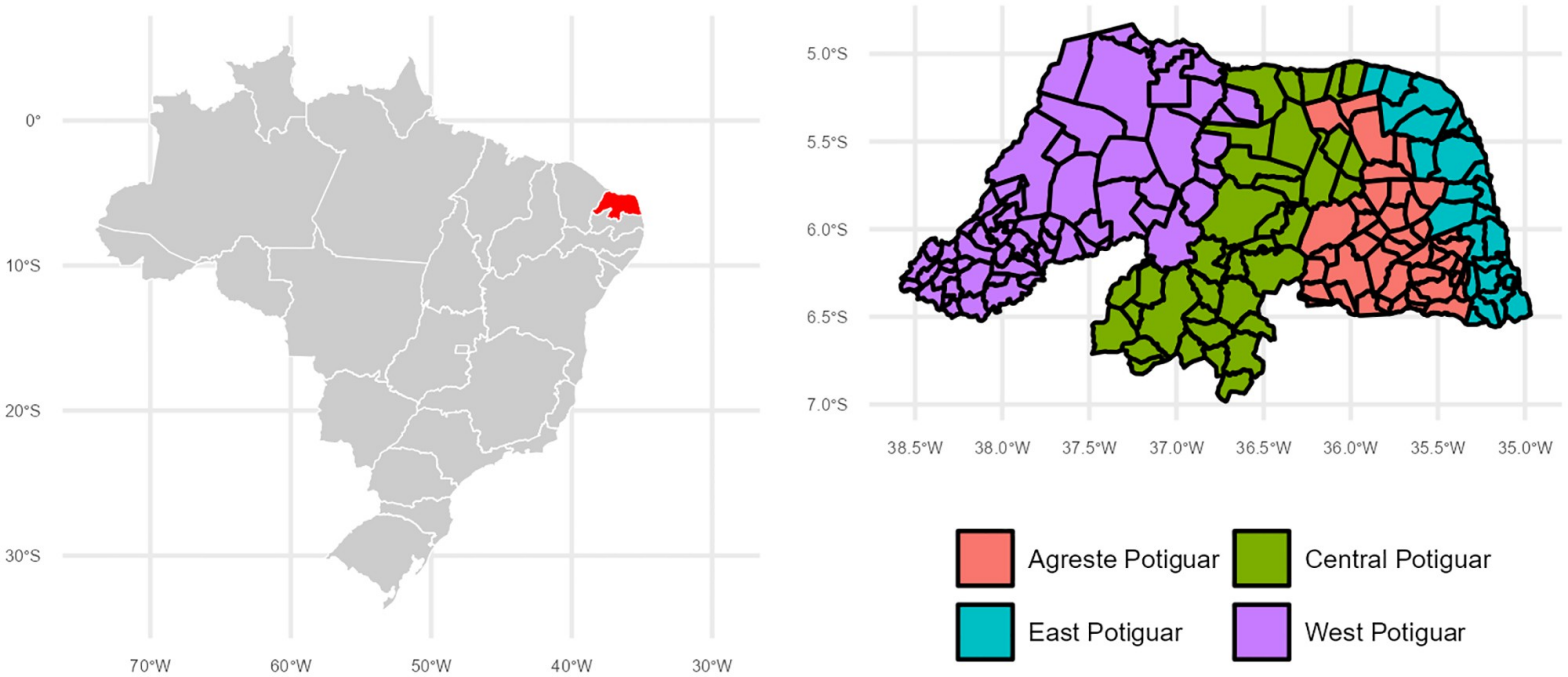
Location and Mesoregions of Rio Grande do Norte. The image on the left highlights the northeastern location of Rio Grande do Norte (in red) within Brazil. The image on the right shows the four Mesoregions in Rio Grande do Norte and municipalities within each region. This figure was created using the geobr package in R [25].

The RN state can be divided into four meso-regions (and the number of municipalities within each) which are West Potiguar (62), Central Potiguar (37), Agreste Potiguar (43), and East Potiguar (25). The meso regions and the municipalities represented within each are displayed in Fig 1. Along with reporting municipality-specific findings, statistical results are summarized for these meso-regions.

### Visceral leishmaniasis data

Visceral leishmaniasis cases in RN between 2007 and 2020 were obtained from passive surveillance conducted by the state of Rio Grande do Norte Health Surveillance Department and the Brazilian Ministry of Health (DATASUS; https://datasus.saude.gov.br/acesso-a-informacao/doencas-e-agravos-de-notificacao-de-2007-em-diante-sinan/). These government health agencies fund VL treatment for those diagnosed with the disease and reported by state agencies. The data analyzed in this study includes patients whose disease status was formally diagnosed via ELISA and/or parasitological confirmation of VL. The data available included the municipality where infection was suspected to have occurred along with the municipality where VL cases were diagnosed. If the municipality of infection was unknown (1042 patients) or municipality of infection was outside of RN (13 patients), the municipality of diagnosis within RN was used as the spatial location in the statistical model. The year attached to each case was the year the Brazilian Ministry of Health was notified of the diagnosed case.

**Fig 2:**
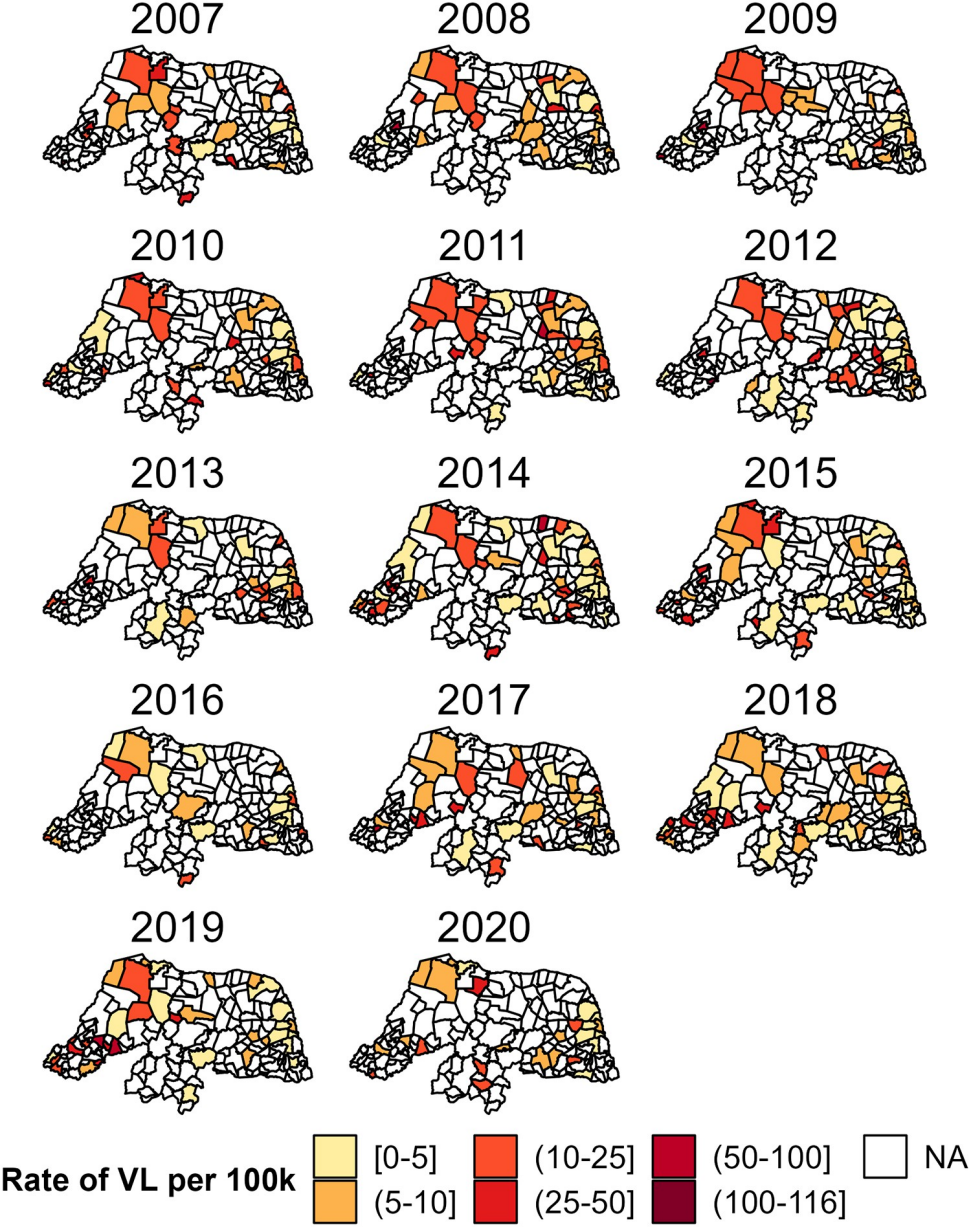
Spatio-temporal distribution of human visceral leishmaniasis rates per 100,000 between 2007 and 2020. This figure was created using the geobr package in R [25].

**Fig 3:**
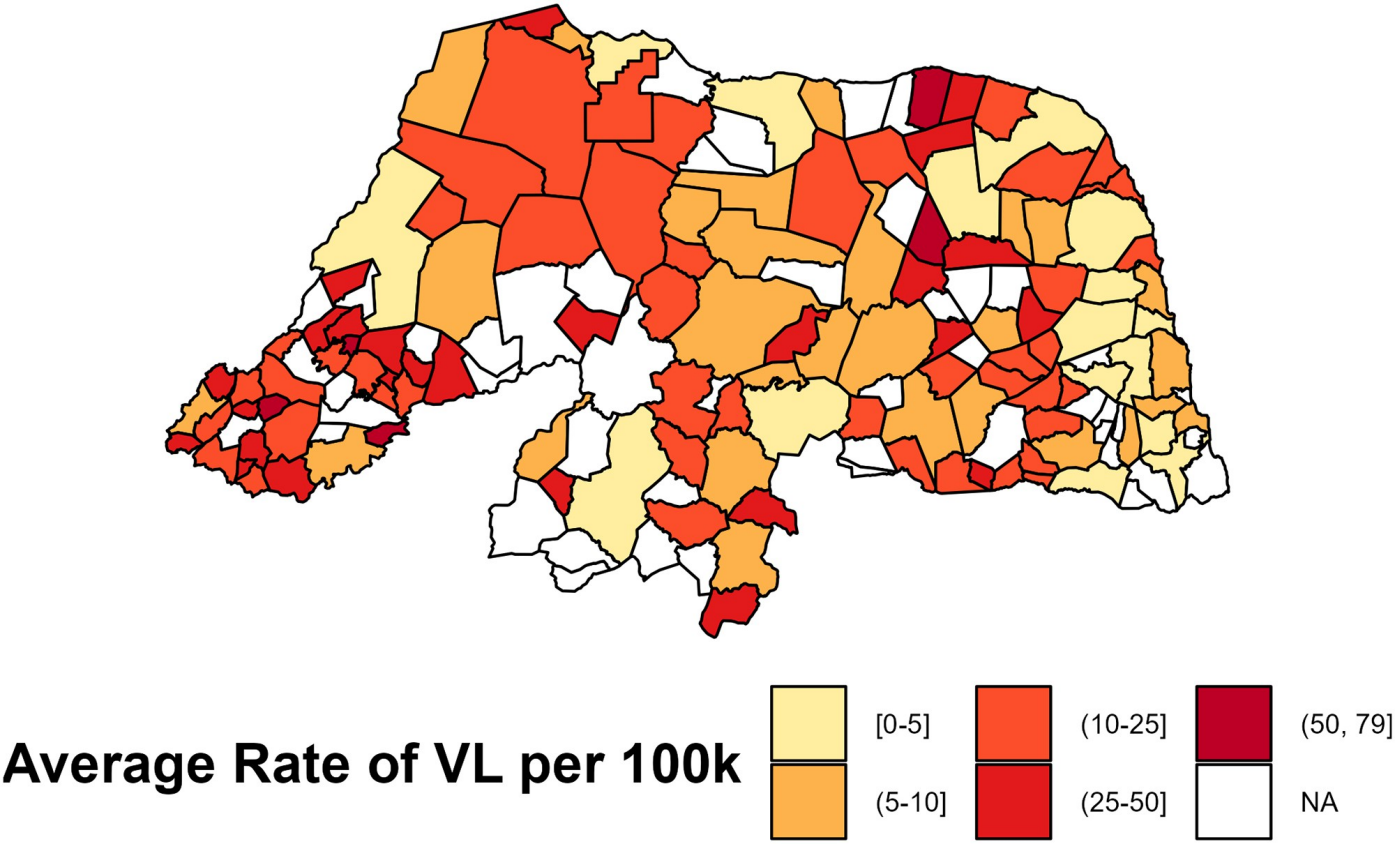
Distribution of average human visceral leishmaniasis rates per 100,000 in Rio Grande do Norte between 2007 and 2020. This figure was created using the geobr package in R [25].

A total of 2,036 VL cases were documented over the fourteen-year study period within 120 of the 167 municipalities in Rio Grande do Norte. Fig 2 shows the yearly rates of VL per 100,000 within each municipality, and Fig 3 features the average rates of VL per 100,000 within each municipality between 2007 and 2020. These figures display the pattern of VL cases across space and over time based on the diagnosed VL cases during the study period. Many municipalities in the West Potiguar mesoregion and some in the East Potiguar mesoregion were observed with large population-standardized VL rates compared to other regions in RN. These municipalities include some of the most populous areas in RN such as Natal, Mossorro, Parnamirim, São Gonçalo do Amarante, Açu, and Extremoz.

### Spatial covariates

Our first research aim was to evaluate VL risk across space and over time in RN. Data representing socioeconomic, environmental and demographic constructs were collected for each municipality from Brazil’s 2010 Census data to quantify regional VL risk throughout RN. Explicit definitions of data collected for the Brazilian Federal Census [26] and the methodology of data collection [27] are provided by the Brazilian Institute of Geography and Statistics.

**Fig 4:**
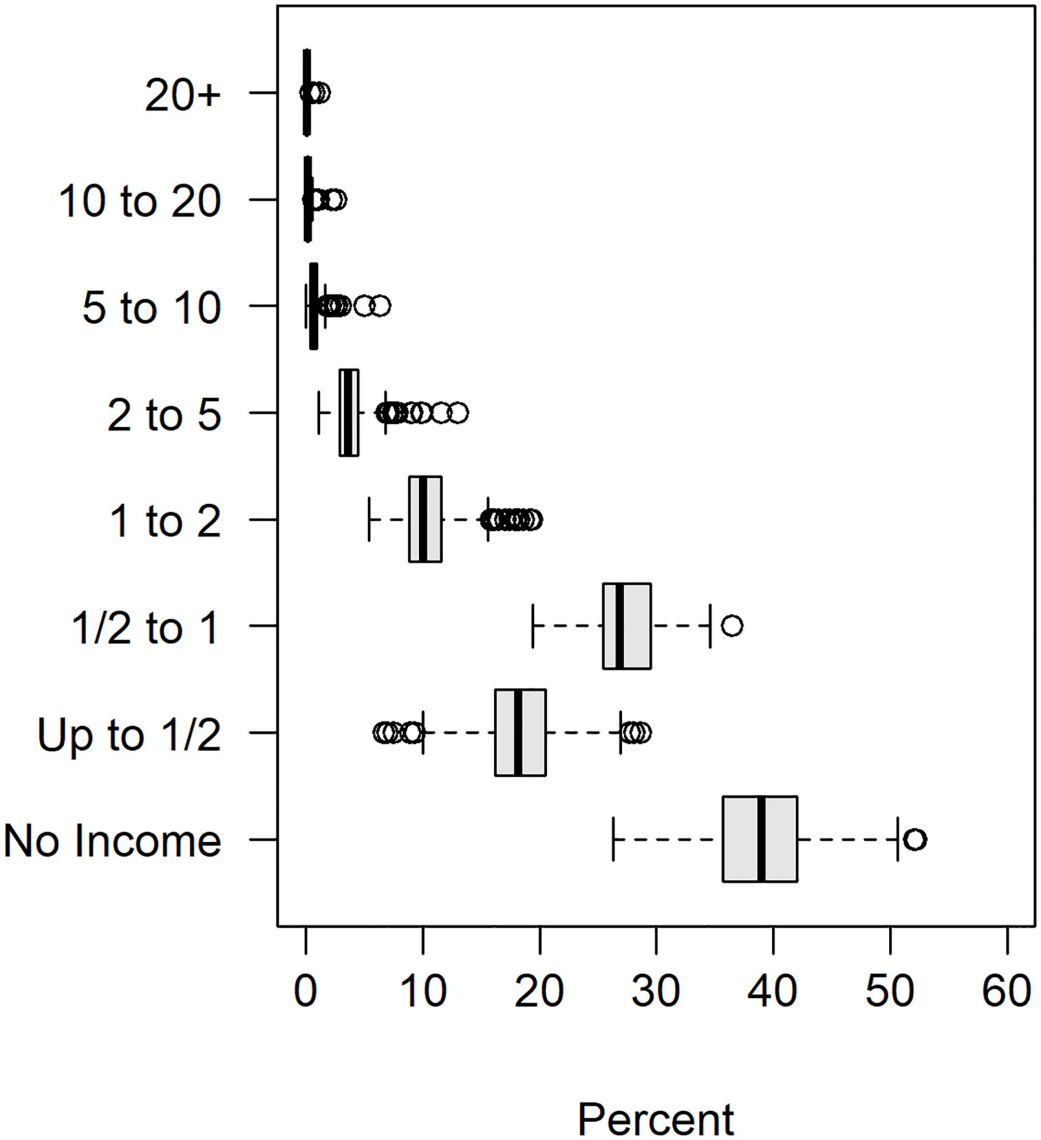
Distribution of percent of residents that fall within each income brackets for all RN municipalities as captured by the 2010 Brazilian Federal Census.

Socioeconomic status of each municipality was quantified by two covariates: the proportion of economically active individuals and a nominal income distribution (for residents above the age of ten). Economically active status was defined for an individual who was employed during the week of data collection [26]. The eight income categories provided by the Census describe proportion of individuals whose monthly income fell within one of the following nominal classes: no income, up 1/2, 1/2 to 1, 1 to 2, 2 to 5, 5 to 10, 10 to 20, or more than 20 times the monthly minimum wage for 2010 which was five hundred and ten reals (510.00 BRL). The no income category included individuals who received only benefits as their income. The nominal income categories, and distribution of percentage of residents within each municipality that fall within those categories, are displayed in Fig 4. Income brackets were combined to provide a three-tier economic distribution within each municipality to describe those with low income (0 to 1 times the minimum income), middle income (1 to 5 times the minimum income), and upper income (5 or more times the minimum income).

**Fig 5:**
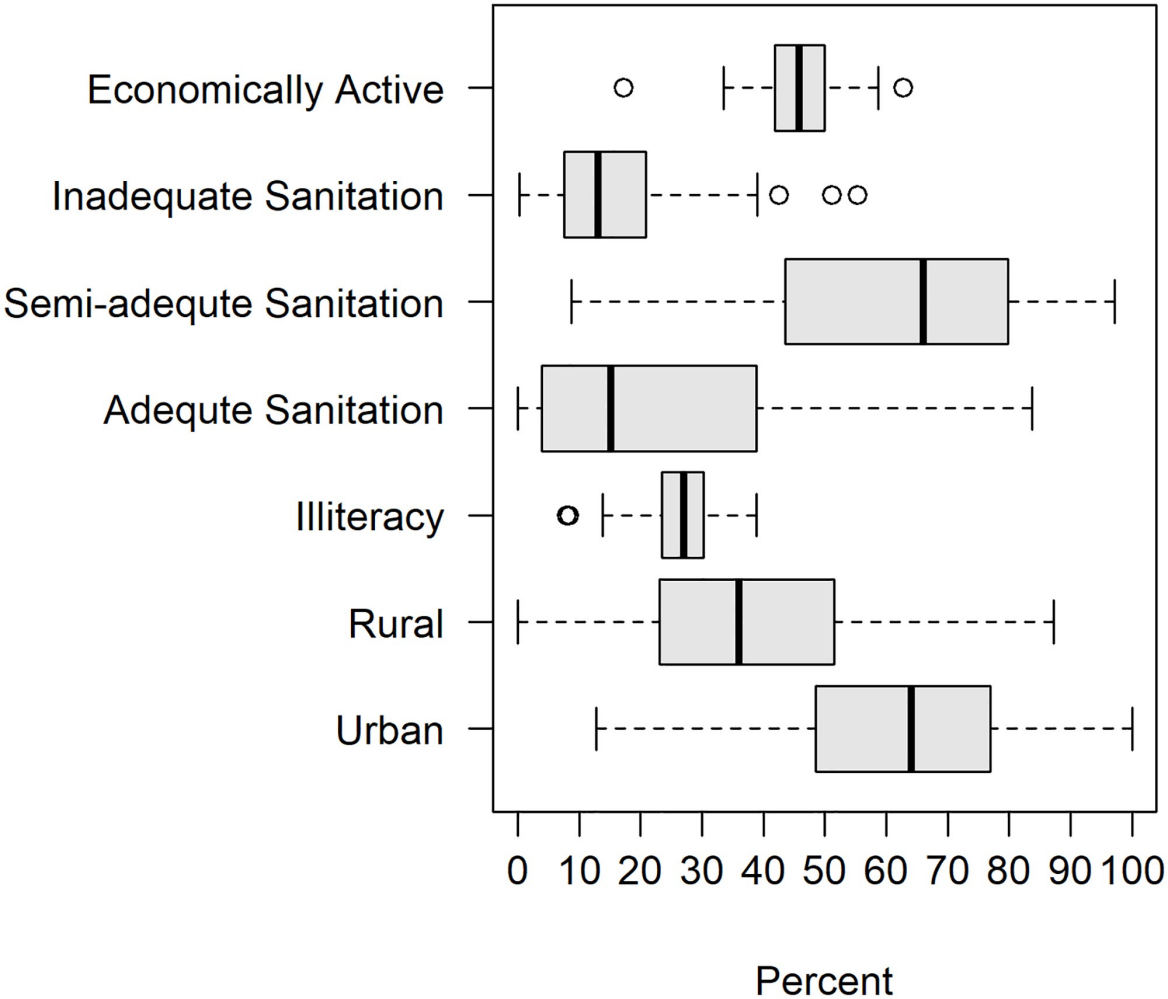
Distribution of percent of residents that were reported as economically active and illiterate within each municipality as captured by the 2010 Brazilian Federal Census. Additionally shown in this figure is the distribution of percent of homes with inadequate or semi-adequate sanitation statues and the percent of homes that were located in rural or urban settings within each municipality.

**Fig 6:**
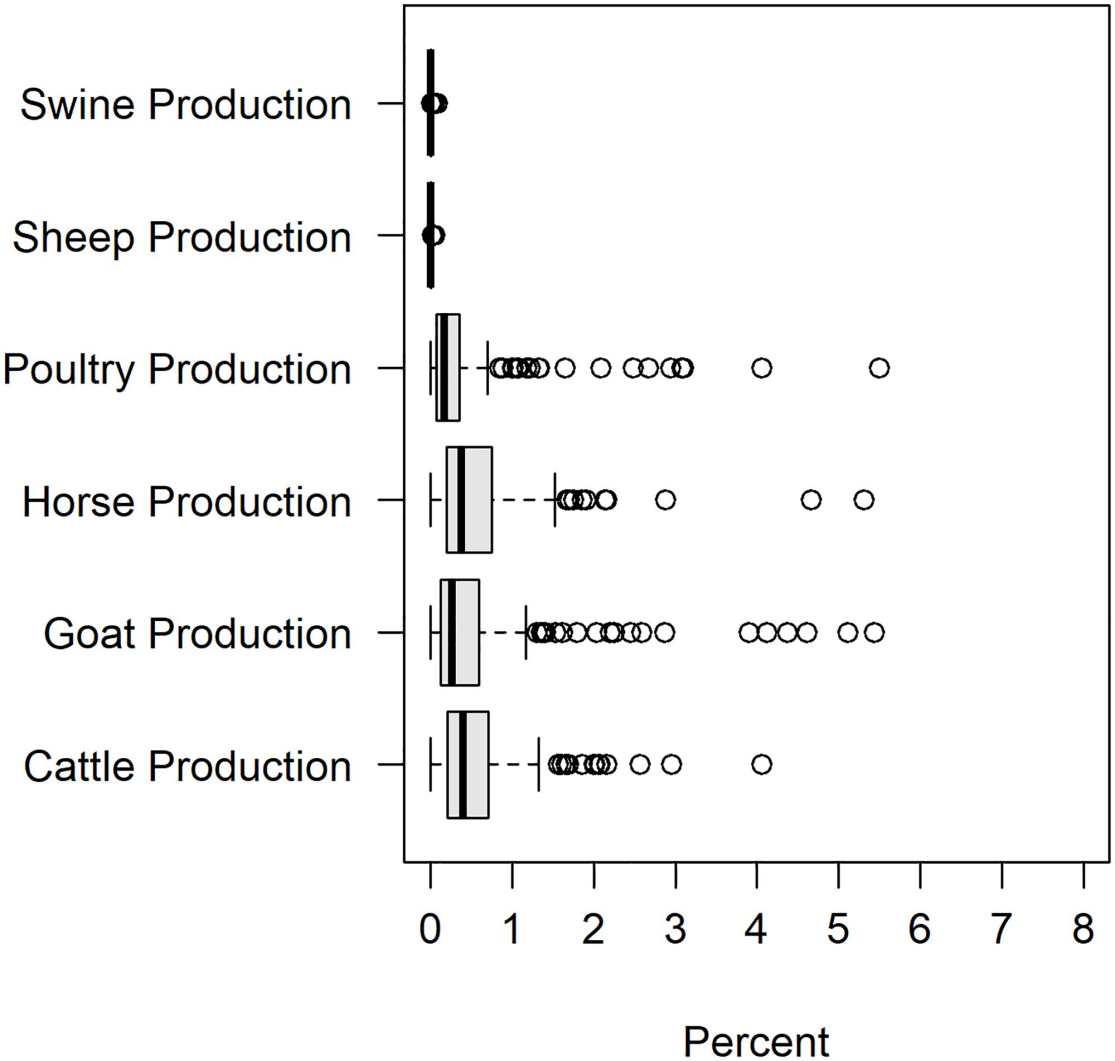
Distribution of percentages of each animal. Two municipalities not plotted in this figure, Mossoro and Parnamirim, had the largest poultry production of all municipalities. The percent of poultry production for both was approximately 16%.

**Fig 7:**
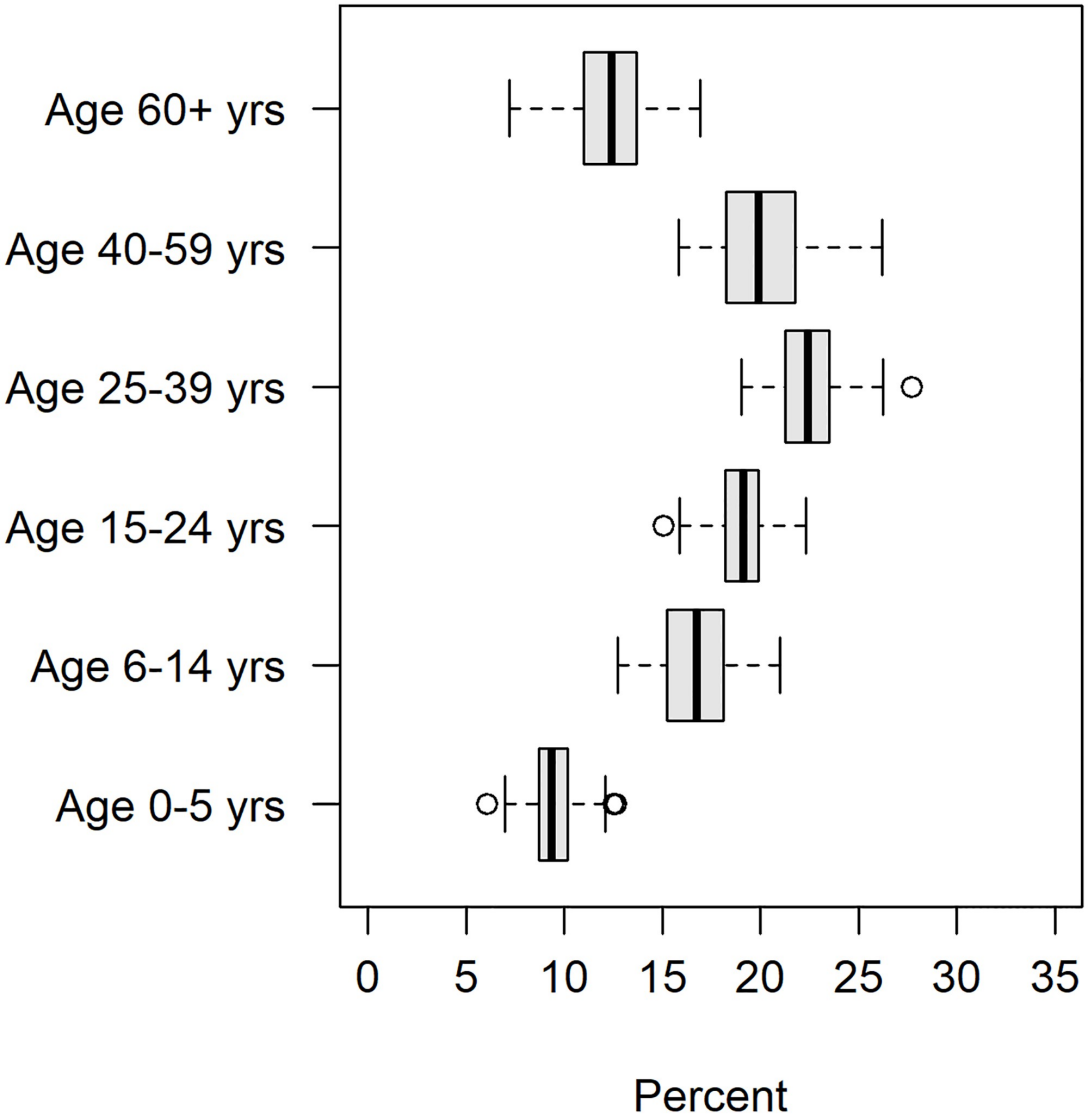
The 2010 Brazilian Federal Census age data was quantified as the percent of individuals within each municipality that fell in each age bracket. The figure above shows the distribution of percentages of residents falling within each age bracket of all RN municipalities.

Covariates characterizing municipality-specific environmental factors included land development (urban or rural), livestock production and level of sanitation of homes (Figs 5 and 6). Livestock production was comprised of large livestock (cattle, horse), midsize livestock (swine, goat, sheep) and small livestock (poultry). Census livestock data at a municipality level informs on the percent of each livestock type that every municipality produces relative to the RN total. Livestock was included to incorporate possible animal reservoirs which may influence the distribution and incidence of VL. Numerous mammals such as chickens [28, 29], swine [30], horses [31, 32] and cattle [10] have been documented as blood sources for sandflies in Brazil and thus serve as possible reservoirs. Livestock production for buffalo and quail were also available but were omitted from the analysis due to a lack of spatial heterogeneity. Households with adequate forms of sanitation had water supplied by the general water distribution network, disposal of sanitary sewage by the general sewage system (or septic tank) and direct or indirect garbage collection. Households missing at least one form of sanitation were considered to have a semi-inadequate sanitation status, while households missing all forms of sanitation were labeled as having inadequate sanitation. Covariates measuring the age distributions for each municipality were also included. Age distribution was quantified using the following age brackets: 0–5 yrs, 6–14 yrs, 15–24 yrs, 25–39 yrs, 40–59 yrs, and 60 years or more (Fig 7). Age distribution was included as a covariate given Brazil’s changing demographics of VL incidence increasing in older adults [15, 33].

### Statistical methods

In this manuscript, we first define the likelihood structure assumed for the observed data from which municipality-specific risk of VL across space and time are estimated. We discuss assumptions on missing data and how our modeling framework works with missing data. Next, we review several modeling structures we considered to account for spatial and temporal heterogeneity across RN. Then, we use an EFA to create interpretable and uncorrelated factor scores that result from common sources of variability in the highly correlated Census data. The resulting EFA factor scores are used as covariates in the model to address the risk of multicollinearity within the statistical model. We justify the need for covariates in the model by fitting models with and without covariate information and comparing model fit by the Deviance Information Criteria (DIC) [34]. The final model combines the EFA results with the best fitting spatial-temporal structure. This resultant model is fit within a hierarchical Bayesian framework where disease risk is estimated in areas where incidence of VL were observed. In areas where incidence of VL was missing, disease risk is predicted based on the observed data. Integrated Nested Laplace Approximation (INLA) methodology was used to obtain approximate Bayesian model inference with this methodology implemented through the R-INLA software [34, 35]. The prior parameters and hyperparameters specification for each model fitted used the default R-INLA specifications.

#### General structure of the hierarchical Bayesian model

The study area and period covered fourteen years across 167 municipalities in RN. We define *i* and *t* to be the municipality and year indices such that *i* ∈ {1, 2, …, 167} and *t* ∈ {1, 2, …, 14}. The Poisson likelihood in Eq 1 was assumed for the visceral leishmaniasis cases in RN in which *Y*_*it*_ and *η*_*it*_ represent the number of VL cases and relative risk (RR) for the *i*^th^ municipality and *t*^th^ year, respectively [36].

$$\begin{array}{c} Y_{it}∼\text{Poisson}\left(E_{it}\times \eta_{it}\right) \end{array}$$ (1)

The focus of this study is to model disease risk by considering municipality risk factor heterogeneity and spatio-temporal patterns as shown in Eq 2. The disease risk parameter (*η*_*it*_), is modeled as a function (*f*) of risk factors (**x**_*i*_), their effect on disease risk (***β***), and a function (*g*) of a vector of random effects (***θ*_*it*_**) capturing spatio-temporal patterns. The functional form of functions *f* and *g* are discussed in future sections.

$$\begin{array}{c} \eta_{it}=f\left(x_{i},\beta \right)+g\left(\theta_{it}\right) \end{array}$$ (2)

Furthermore, *E*_*it*_ represents the expected number of cases for the *i*^th^ municipality and *t*^th^ year in the event the *i*th municipality’s risk of disease is unaffected spatially or temporally. This yearly area-specific expected quantity can be calculated as a weighted average of the prevalence of the disease (*p*) and the area’s population that year (*n*_*it*_). The yearly population for each municipality is estimated by the Brazilian Institute of Geography and Statistics (IBGE) while the prevalence of VL can be estimated as the ratio of total VL cases in RN and the overall RN population total. The yearly prevalence estimate would require the observation of all VL cases in RN each year. However, as shown in Figs 2 and 3, VL cases were not documented for all municipalities in RN each year. We avoid making the restrictive assumption that missing information implies absence of disease, as there could be individuals infected with VL that went undiagnosed during the study period.

Therefore, we establish a baseline prevalence estimate using the complete data. Let A={i,j} be the set of location/time indices with observed data such that *Y*_*it*_ and *n*_*it*_ for i,t∈A correspond to the number of observed VL cases and the population estimates for municipalities with observed VL cases. Based on these observed values, we can calculate a naive estimate of VL prevalence ($\hat{p}$) in RN as a ratio of the total observed VL cases and the total population estimates of municipalities with observed cases. We can also obtain a naive estimate of the yearly expected number of disease cases for location *i* at year *t* (*E*_*it*_). This expected measure can be calculated as a product of the naive prevalence estimate weighted by the location population estimate that year (Eq 3).

$$\begin{array}{c} \begin{array}{cc} E_{it} & =n_{it}\;*\;\hat{p}\\ & =n_{it}\;*\;(\frac{\sum_{t\in A}\sum_{i\in A}Y_{it}}{\sum_{t\in A}\sum_{i\in A}n_{it}}) \end{array} \end{array}$$ (3)

The hierarchical Bayesian model was adopted to allow for flexible modeling of area-specific relative risk in which a linear function of a set of fixed effects and a corresponding set of random effects contribute to the modeling of the risk outcomes. The random effects structure served to explain spatio-temporal patterns of relative risk, whereas the fixed effects serve to explain spatial risk variation potentially driven by municipality-specific risk factors. Furthermore, this modeling framework allows for the prediction of disease risk for areal units with missing data by utilizing the estimated posterior distribution of disease risk parameters. Therefore, given the spatio-temporal nature of VL and many municipalities not reporting VL cases in multiple years, a hierarchical Bayesian model was adopted to estimate and predict visceral leishmaniasis risk in RN between 2007 and 2020 more accurately.

#### Spatio-temporal structure selection

Fitting a Bayesian hierarchical model allowed for flexible specifications of the random effects to explain spatio-temporal patterns of VL risk. Shown below are the five (M1–M5) candidate models with fixed effects structure *f*(**x**_*i*_, ***β***) = *β*_0_ and differing random effects structures *g*(***θ*_*it*_**) considered.

$$\begin{array}{c} log(\eta_{it})=\beta_{0}+\phi_{i} \end{array}$$ (M1)

$$\begin{array}{c} log(\eta_{it})=\beta_{0}+\phi_{i}+\psi \times t \end{array}$$ (M2)

$$\begin{array}{c} log(\eta_{it})=\beta_{0}+\phi_{i}+\left(\psi +\delta_{i}\right)\times t \end{array}$$ (M3)

$$\begin{array}{c} log(\eta_{it})=\beta_{0}+\phi_{i}+\gamma_{t}+\omega_{t} \end{array}$$ (M4)

$$\begin{array}{c} log(\eta_{it})=\beta_{0}+\phi_{i}+\gamma_{t}+\omega_{t}+\delta_{it} \end{array}$$ (M5)

Model M1, the base candidate model, was a scaled version of the Besag-York-Mollié (BYM) model that accounts for between and within-municipality spatial variability through a spatial random effect (*ϕ*_*i*_) [37]. In the BYM model, the spatial random effect promoting smooth area relative risk estimates is defined as $\phi_{i}=u_{i}^{*}+v_{i}^{*}$; the sum of a spatially structured random effect ($u_{i}^{*}$) and a unstructured spatial random effect ($v_{i}^{*}$). The spatially structured random effect follows a normal prior centered at the average spatial effects for surrounding municipalities and variance weighted by the number of neighboring areas. Therefore, the spatially structured random effect accounts for similarities in disease outcomes between neighboring areas. The unstructured random effect accounts for independent municipality-specific non-spatial heterogeneity and follows a zero-mean normal prior with variance $\sigma_{v^{*}}^{2}$. A mathematical representation of the priors for the BYM model parameters is specified below where N(i) indexes the municipalities that share boundaries with the *i*th municipality and #N(i) is the number of municipalities that share boundaries with the *i*th municipality.

$$\begin{array}{c} v_{i}^{*}∼Normal(0,\sigma_{v^{*}}^{2}) \end{array}$$

$$\begin{array}{c} u_{i}^{*}|u_{k\neq i}^{*}∼Normal(\frac{\sum_{k\in N(i)}u_{k}^{*}}{\#N(i)},\frac{\sigma_{u^{*}}^{2}}{\#N(i)}) \end{array}$$

The scaled Besag-York-Mollie model (BYM2), discussed in [38], is a reparameterized version of the BYM model where the spatial random effect (*ϕ*_*i*_) is a weighted average of the structured and unstructured components. Specifically, $\phi_{i}=\frac{1}{\sqrt{\tau }}(v_{i}\sqrt{1-\lambda }+u_{i}\sqrt{\lambda })$ where *u*_*i*_ and *v*_*i*_ are respectively similar to $u_{i}^{*}$ and $v_{i}^{*}$ in the BYM model but are standardised to have generalised variance equal to one [34, 35]. In the BYM2 model, *τ* represents the marginal precision contribution from *u*_*i*_ and *v*_*i*_ while λ is a mixing parameter representing the percent of marginal variation explained by the spatially structured random component. Scaling of the BYM model allows for a uniform interpretation of the marginal variance across varying spatial structures and enables easier specification of prior distributions for BYM2 model parameters [38]. Default INLA Penalized Complexity (PC) priors for log transformed *τ* and logit transformed λ hyperparameters were used. PC priors were introduced by [38]. The mathematical expression of PC priors for the BYM2 model is thoroughly outlined in [38].

$$\begin{array}{c} \text{log}(\tau )∼\text{PC}(1,0.01) \end{array}$$

$$\begin{array}{c} \text{logit}(\lambda )∼\text{PC}(0.5,0.5) \end{array}$$

The second candidate model (M2) adds a global linear time effect (*ψ*) to explain the yearly trend in VL risk for the state of Rio Grande do Norte between 2007 and 2020. The time effect was given a default normal prior with mean and precision parameter of 0 and 0.001, respectively. Model M3 adds to model M2 by including an area-specific linear time interaction effect (*δ*_*i*_), as introduced by [39], to account for the linear difference in risk over time for the *i*^th^ area relative to the global RN trend. A negative interaction term (*δ*_*i*_ < 0) indicates the municipality-specific trend that is less steep than the overall RN temporal trend. A positive interaction term (*δ*_*i*_ > 0) implies a steeper municipality-specific risk trend compared to the overall RN trend. The log transformed area-specific trend was given a log gamma prior with shape parameter of one and rate (or inverse-scale parameter) of 0.00005. The log gamma prior on the log transformed parameter of interest is equivalent to placing a gamma prior with the same shape and rate parameters on the parameter of interest.

The linear effects of time on the log transformed risk of VL, as specified in models M2 and M3, may not be a reasonable assumption. Therefore, models M4 and M5 specified a temporally structured effect using a Gaussian random walk model of order 1 (*γ*_*t*_) and an overall unstructured time effect (*ω*_*t*_), respectively, as seen in [40]. The overall time effect had a normal prior centered at zero and a precision hyperparameter that followed a gamma prior with shape parameters of 1 and rate parameter of 0.00005. Model M5 expanded model M4 to include an unstructured space and time interaction parameter (*δ*_*it*_) that adjusts for spatio-temporal variation in the data unaccounted for the main space and time effects [40]. Knorr-Held [40] propose other ways in which this interaction can be structured based on various assumptions. The *δ*_*it*_ parameter was given a normal prior with precision hyperparameter following a gamma prior with default shape and rate parameters of 1 and 0.00005 respectively.

#### Fixed effects structure

Risk factors obtained from the 2010 Census jointly account for social, economic and environmental differences between municipalities. Empirical Spearman’s correlation coefficients for social vulnerability indices derived from RN’s 2010 Census data show correlation estimates ranging between 0.33 and 0.55 (S2 Fig). We conduct an EFA to preserve the interpretability of covariate effects on disease risk estimates while mitigating correlation between the Census data. An EFA is a dimension reduction technique that helps uncover underlying relationships between large number of correlated variables [41]. Factor analysis techniques require verification that the observed data is suitably intercorrelated. To assess adequacy of the 2010 IBGE Census data for Exploratory Factor Analysis, Bartlett’s test of Sphericity [42] and Kaiser-Meyer-Olkin (KMO) statistics [43] were jointly considered. In this manuscript, we utilize an EFA using the R package psych [44] to replace the correlated Census information, as seen in S1 Fig, with the resulting factor scores in the statistical model from which we draw inference. The EFA conducted assumed a linear correlation between the Census variables and used a principal factor extraction method to obtain the factor scores. To mitigate correlation between the resulting factor scores and address risk of multicollinearity within the statistical model, a varimax rotation assuming orthogonality between the latent variables was used. Categorical variables describing within-municipality characteristics (i.e. all covariates except livestock variable) provide information regarding 100% of the municipality. Therefore, for a categorical variable, *k* − 1 categories were included in the factor analysis since the *k*^*th*^ category can be described as a linear function of remaining *k* − 1 categories.

The resulting *p* factor scores served as the covariates in the model to inform on municipality risk factor heterogeneity. To access adequacy of adding covariates to the spatially and temporally structured candidate models, we additionally fit models M1–M5 with linear combination of the factor scores *f*(**x**_*i*_, ***β***) = *β*_0_ + **x**_*i*_***β***. In the covariate structure, **x**_*i*_ denotes the 1 × *p* vector of factor scores for municipality *i*. For all models fitted with covariates, the fixed effects coefficients (***β***) were given a normal distribution prior with mean parameter zero and precision of 0.001.

## Results

### Spatial and temporal model structure

DIC results used to compare candidate models considered are seen in Table 1. Model M1, which assumed no temporal trend on disease risk, had the highest DIC results. Models M2 and M4, which assumed change in risk of VL over time was the same for each municipality, had the next highest DIC results. The remaining two models, Models M3 and M5, had the lowest DIC results. While Model M3 allowed for changes in VL risk over time to vary between municipalities, it assumed changes in VL risk were constant over time, whereas Model M5 allowed for non-constant changes of VL risk each year for each municipality. The added flexibility provided by Model M5 fit the data best indicated as the model with the lowest DIC.

**Table 1:** Model Fit DIC Statistics.

Model	without covariates	with covariates
M1	1641.82	1625.05
M2	1640.35	1622.98
M3	1538.93	1525.90
M4	1565.72	1552.00
M5	1508.51	1494.16

### Exploratory factor analysis

Factor analysis tools assume there is a set of latent variables that give rise to the observed set of correlated data. Bartlett’s test using the Census variables resulted in statistically significant test (*p* − *value* < 0.001). A KMO statistic of 0.78 was observed for the overall Census variables with all single-item KMOs greater than or equal to 0.69. The joint results for Bartlett’s test of Sphericity and Kaiser-Meyer-Olkin statistics indicate the Census data was adequate for factor analysis.

**Fig 8:**
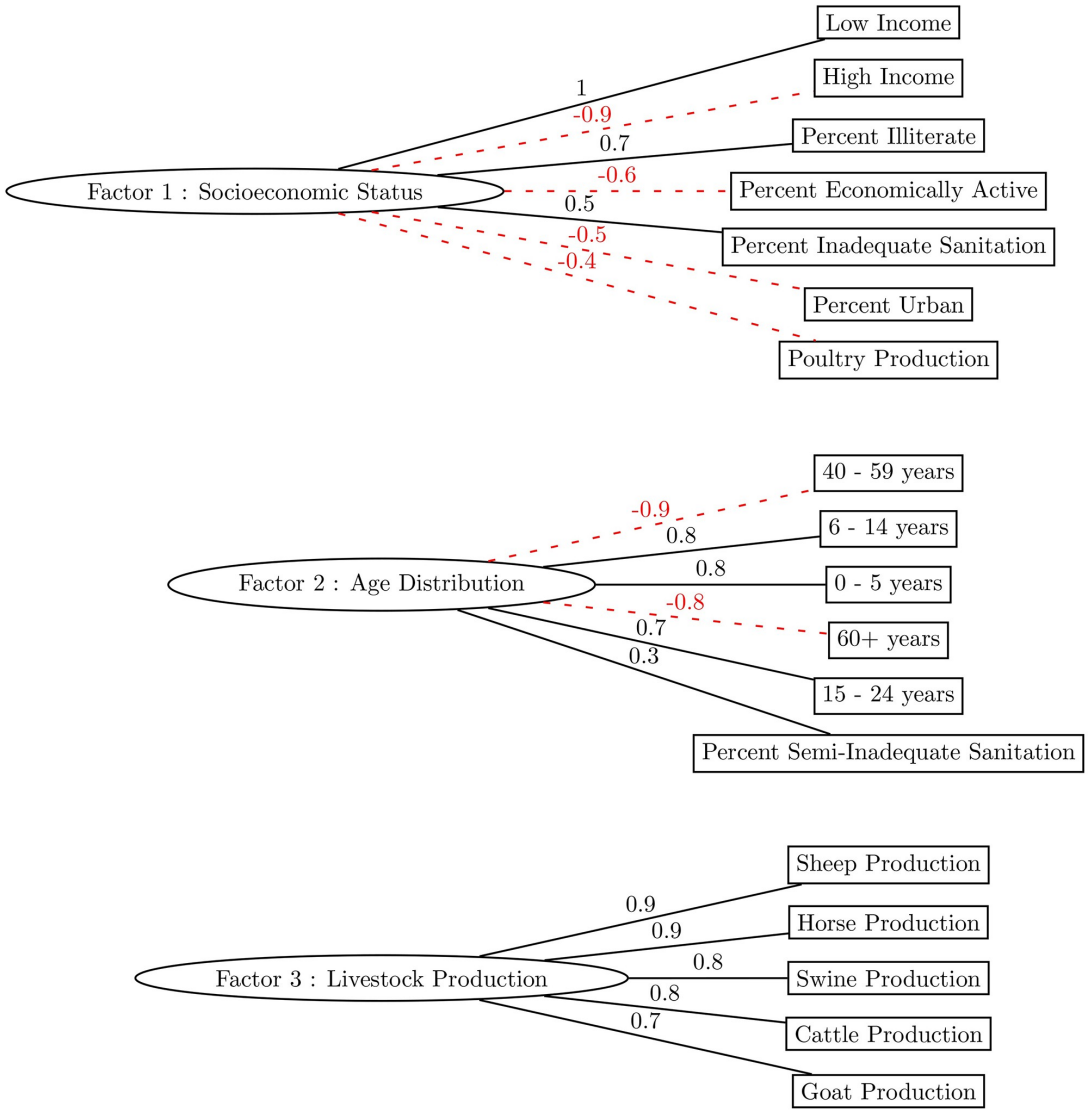
Human VL Exploratory Factor Analysis results with 2010 Brazilian Federal Census primary variable contributions.

The number of factors to extract from the EFA analysis were obtained by examining the scree plot which was estimated using parallel analysis. The scree plot suggested extracting three factors representing the latent relationships among the Census variables. Fig 8 shows the predominant relationship (negative or positive) of the Census variables towards each factor. The relationship between Census data and the resulting factors is represented by the EFA factor loadings which are provided in more detail in S1 Table.

Factor 1 was primarily made up of economic, education, and environmental variables. Factor 2’s main contribution was age distribution within municipality, and Factor 3 was predominantly measured by the degree of municipality livestock production relative to the overall production in Rio Grande do Norte. Therefore, in labeling the factors resulting from the EFA analysis, Factor 1 represents the Socioeconomic Status of a municipality, Factor 2 represents the Age Distribution of a municipality and Factor 3 measures Livestock Production of a municipality.

DIC measures for models M1–M5 fitted with the resulting factor scores as covariates can be found in Table 1. Adding covariate information improves model fit for the spatially and temporally structured candidate models based on DIC measures which provides evidence that the covariate information helps to explain variability in disease risk. Model M5 with covariates has the lowest DIC measure of all models fitted. The results for this best fitting model are further dissected in the following sections.

### Final model inference

The best fitting model to evaluate risk of disease across Rio Grande do Norte between 2007 and 2020 was a log-linear Poisson hierarchical model (M5). In this model, the log-transformed relative risk structure for municipality *i* at time *t* is a function of a linear combination of fixed effects *f*(**x**_*i*_, ***β***) = (*β*_0_ + **x**_*i*_***β***) and a linear combination of random effects *g*(***θ*_*it*_**) = (*ϕ*_*i*_ + *γ*_*t*_ + *ω*_*t*_ + *δ*_*it*_). In the fixed effects structure, **x**_*i*_ = [*x*_1*i*_, *x*_2*i*_, *x*_3*i*_] represents the first, second, and third EFA factor scores for location *i* while ***β*** = [*β*_1_, *β*_2_, *β*_3_] is the vector of coefficients to estimate in addition to the intercept *β*_0_ parameter. For the random effects structure, the location-specific spatially structured and unstructured random effects are denoted as *u*_*i*_ and *v*_*i*_, respectively. The *γ*_*t*_ and *ω*_*t*_ parameters in the model denote the yearly change in VL risk estimated for all municipalities. Risk trends are allowed to vary each year within a municipality through the *δ*_*it*_ parameter which increases or decreases the trend each year as necessary based on the observed data.

We estimated posterior means, 95% Highest Posterior Density (HPD) credible intervals, and posterior probabilities which summarize the posterior distributions of parameters of interest. Posterior distributions help quantify our uncertainty about the parameters of interest given the observed data. Positive covariate parameters would result in higher VL risk estimates while negative covariate parameters would lead to lower VL risk estimates.

**Fig 9:**
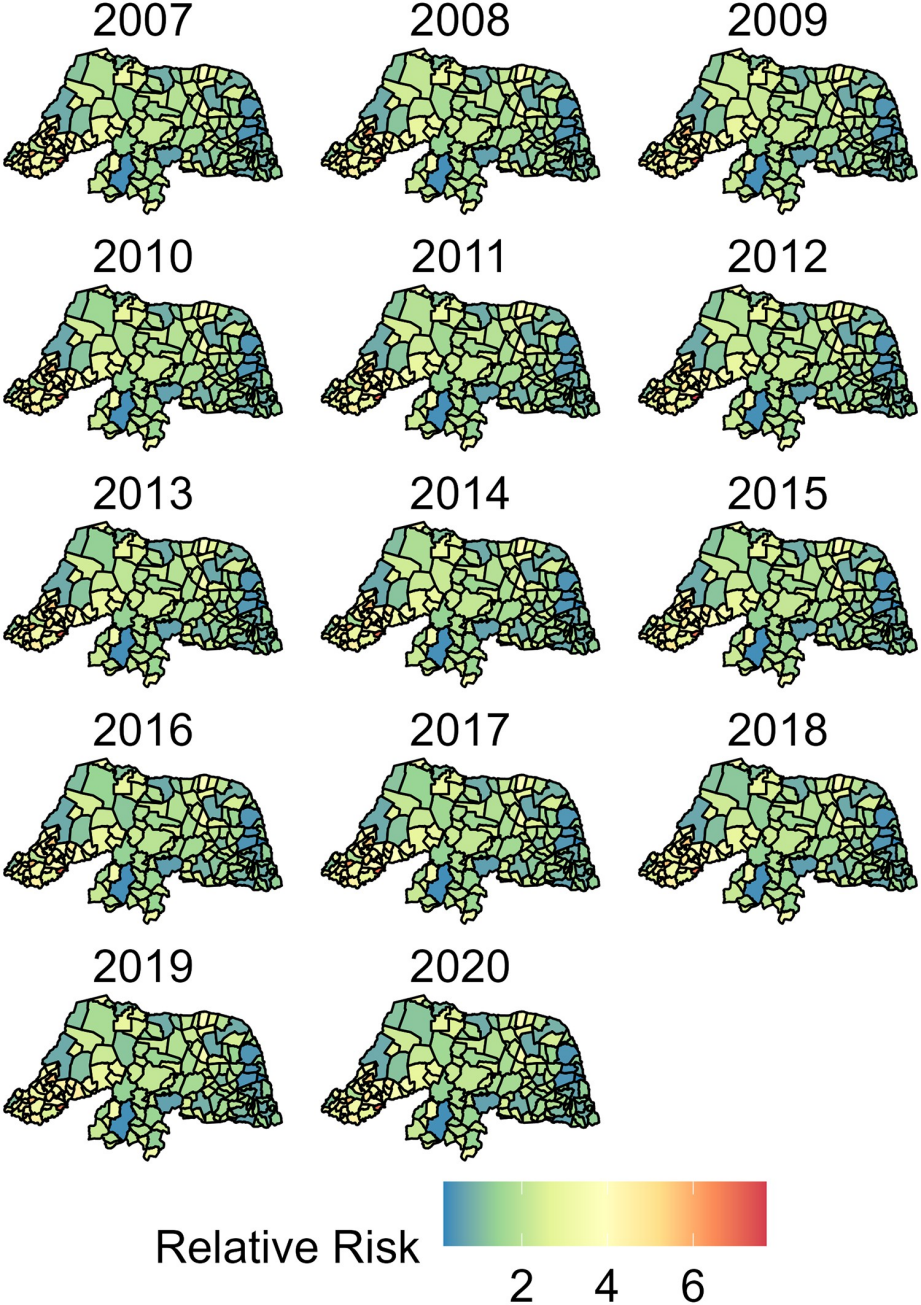
Spatial and Temporal posterior mode estimate of municipality-level relative risk of disease. This figure shows regions where, based on model results, it is likely that the risk of disease is higher than what we deem clinically significant. This figure was created using the geobr package in R [25].

**Fig 10:**
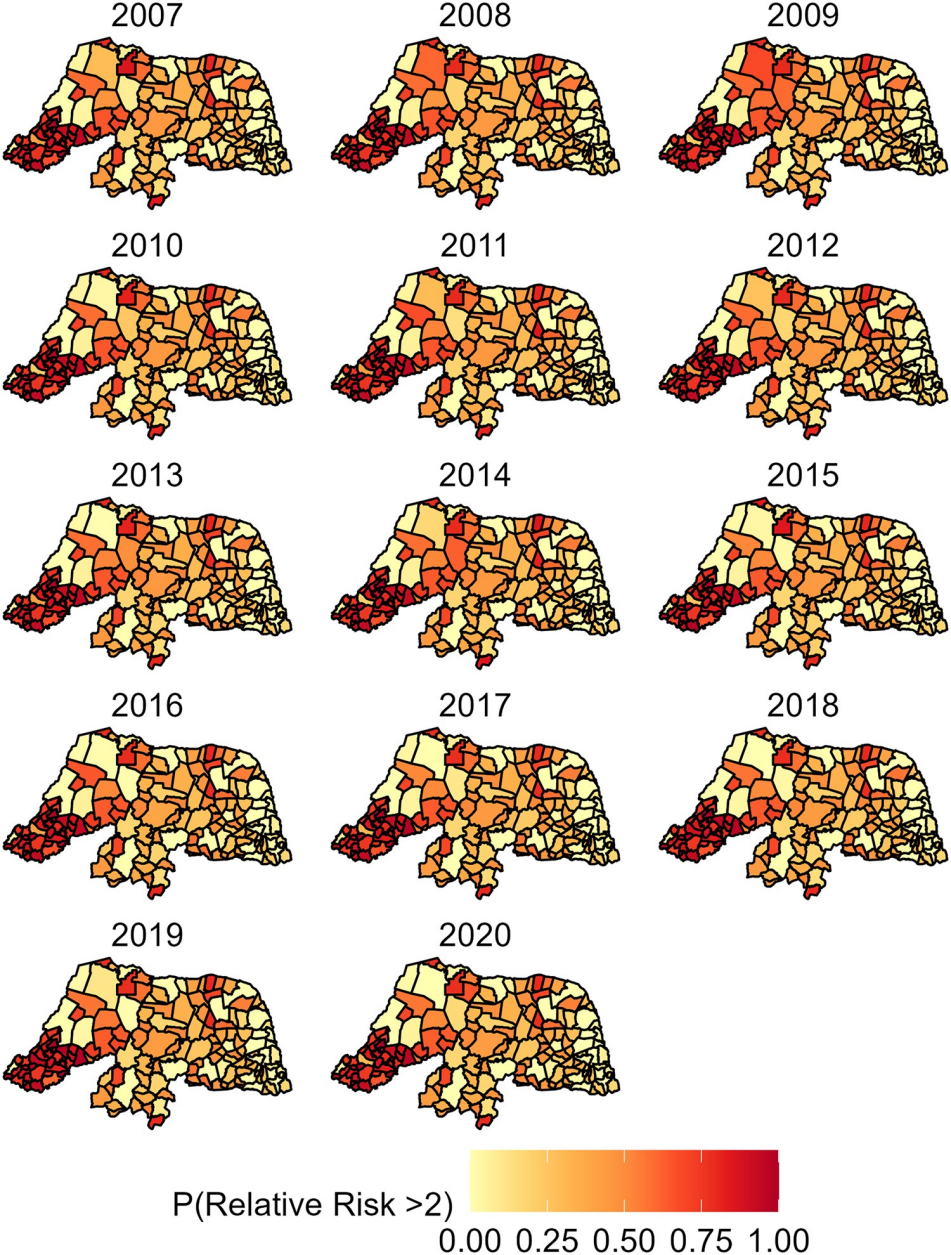
The spatial and temporal posterior probability that a municipality’s relative risk estimate shown in Fig 9 is greater than two. Darker shades of red indicate it is likely that the municipality’s relative risk estimate is clinically significant. This figure was created using the geobr package in R [25].

The visceral leishmaniasis RR estimates after adjusting for the EFA factor score (*η*_*it*_) were used to answer the first research aim to evaluate disease risk in RN across space and over time. Based on the model structure, a RR equal to one indicates the risk of the average municipality in RN. Relative risks greater than two indicate more than double the risk of VL than what would be expected if disease risk was not spatially or temporally different in RN. A RR more than double what would be expected was deemed to be clinically significant (i.e. high-risk). Fig 9 shows the VL RR across RN over time. Fig 10 displays the exceedance probabilities that the RR estimates surpass a value of two which, by our definition, would be deemed clinically significant. Exceedance probabilities provide strength of evidence towards clinically significant or high-risk regions. In Fig 10, areas with high probability indicate there is strong evidence that these are high-risk regions which could benefit from future interventions and resources to address local disease prevalence. We also include S3 Fig to show exceedance probabilities of the RR estimates greater than one. This additional figure highlights municipalities that, given the data, are likely to have risk of VL greater than what would be expected if disease risk was not spatially or temporally different S3 Fig.

**Fig 11:**
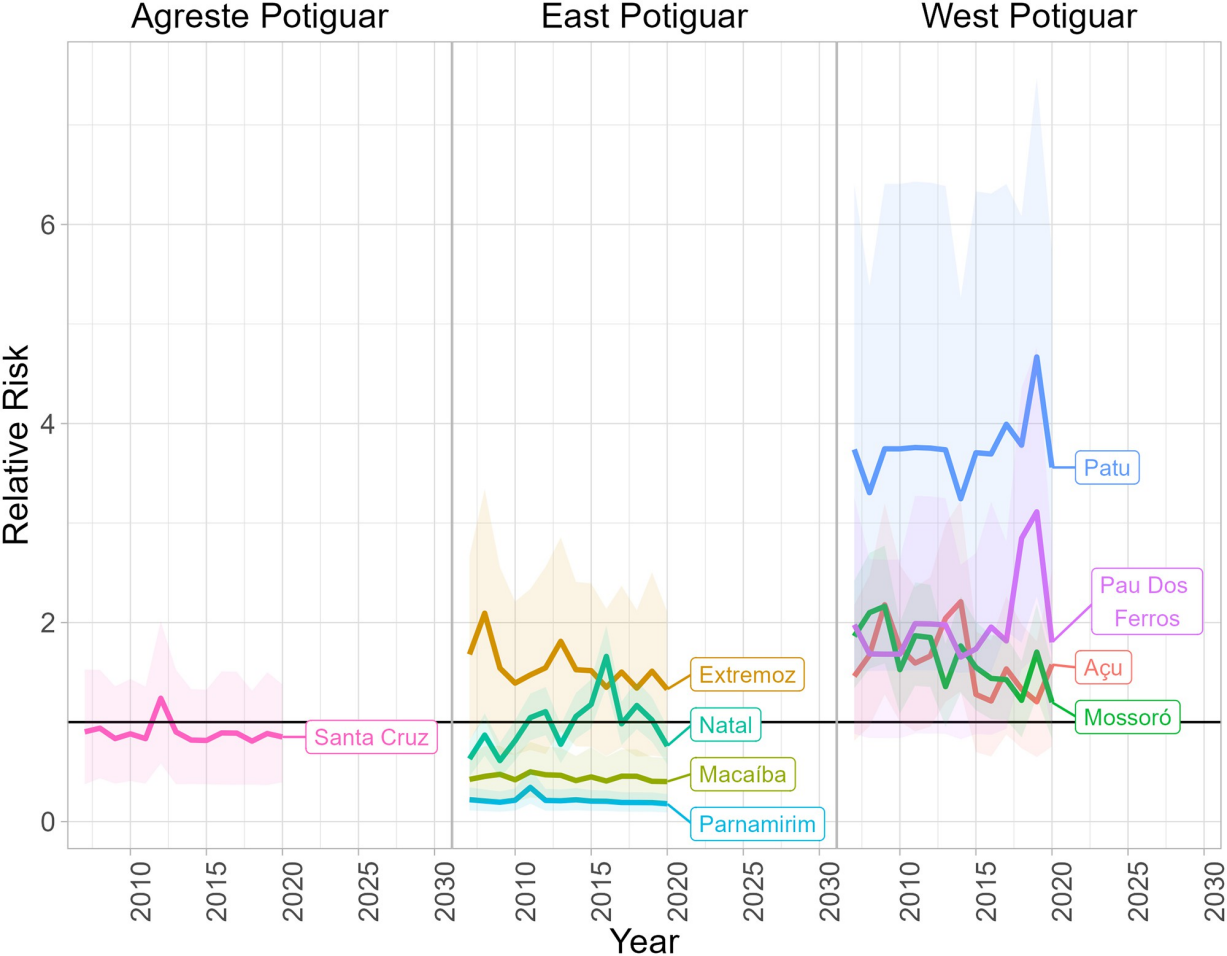
This figure shows the most varying municipality-specific RR trends within the four mesoregions in RN. Municipality-specific risk trends were obtained as posterior distribution estimates if VL cases were observed or as predictions from the posterior predictive distributions if VL cases were unobserved for each year. Intervals included in this figure for each trend are 95% HPD credible intervals. The horizontal line represents a relative risk of one.

Relative risk trends by mesoregions for all municipalities can be seen in S4 Fig. For most municipalities, regardless of mesoregion, the RR trends were relatively consistent over the study period. S4 Fig shows few municipalities had RR trends that varied greatly over time. To identify which municipalities had large changes in risk, the variability in yearly percentage change of RR relative to the prior year was calculated. Risk trends for municipalities within the top 5% highest variability in yearly change of risk are shown in Fig 11. This figure allows for a direct comparison of changes in VL RR trends within and between mesoregions for the highest varying municipalities.

Table 2 informs on the posterior mean and 95% HPD credible intervals for the EFA factor scores coefficients estimated. The posterior probability that an effect is positive is displayed in the last column of the table to quantify the model’s uncertainty of the associations between the Socioeconomic, Age and Livestock factor scores and disease risk estimates.

**Table 2:** Posterior distribution summaries for fixed effect parameters (*β*).

Fixed Effects	*β*	Posterior Mean	95% Credible Interval	P(*β* > 0)
Intercept	*β*_0_	0.4829	(0.3449, 0.6403)	1
Socioeconomic Status	*β*_1_	0.2372	(0.1151, 0.3590)	0.9999
Age Distribution	*β*_2_	-0.0126	(-0.2033, 0.1795)	0.4429
Livestock Production	*β*_3_	-0.2230	(-0.3383, -0.1089)	0.0001

## Discussion

Municipalities with poor socioeconomic characteristics were reflected through high EFA Socioeconomic factor scores. Characteristics denoting a poor socioeconomic status included high percent of illiteracy, homes with inadequate sanitation, rural surroundings, low-income population, and economically inactive population. Municipalities with opposing characteristics would have lower Socioeconomic factor scores. The posterior mode estimate of the Socioeconomic Factor was positive, further indicating that socioeconomic status was positively related to relative risk of VL. A municipality that only differs by one unit less in the Socioeconomic Factor from another would be estimated to have a lower relative risk of VL (by about 26%) compared to the municipality with the higher Socioeconomic Factor score. It is also estimated that there is a 99.99% probability that there is a positive relationship between poor socioeconomic status and higher risk of VL. The connection between socioeconomic status and risk of VL in Brazil is strongly supported by prior literature [22, 45, 46]. Mediating factors such as sanitation [47, 48] and literacy [4, 11] contribute to risk in the same direction as the present analysis.

A municipality’s contribution to the overall RN Livestock was a characteristic emerging from the EFA that described potential environmental risks of VL. Municipalities that were large producers of sheep, horses, swine, cattle or goats had higher Livestock Production factor scores. There was a negative association estimated for the Livestock factor score and risk of VL. Specifically, a municipality with a one unit increase in livestock production factor score would be estimated to have approximately 20% lower risk of VL compared to a municipality that has a lower production levels. The model estimated a 99.99% probability that there is a negative association between higher livestock productions and lower relative risk of VL. Previous studies have also reported this association of large livestock acting as protective agents against VL, though with varied working hypotheses [49–52]. On one hand, owning large livestock such as cattle may function as a sign of better nutritional status of those residents, thereby providing for overall better immune status and a proxy for overall better health augmenting their immune systems against VL [49–51]. On the other hand, the presence of cattle sheds could promote breeding sites for sandflies [51, 52]. Additionally, it is worth noting that the Livestock Production factor is not directly measuring density of animals and their coexistence with human populations. Instead, this variable could be a proxy for a municipality’s economic productivity representing industrial areas that might be more economically distinct that other areas. This means highly productive areas could be characterized with a higher economic status that might explain the association between livestock production and VL risk estimated by the model. More research is needed to confirm these hypotheses in Northeastern Brazil.

The EFA’s third construct uncovered from the observed Census data described the age distribution within a municipality. Municipalities with high Age Distribution factor scores had a larger percentage of individuals twenty-four years age or younger and larger percentage of households with semi-inadequate sanitation compared to areas with opposite characteristics. The results of the association between the Age distribution factor scores and the risk of VL were inconclusive as the posterior mean of this effect was small and close to zero. Furthermore the probability that the association between the Age distribution factor scores and the risk of VL was positive or negative was approximately the same. Although VL has historically been mostly identified in children, recent studies support that the average age of VL cases diagnosed has increased which may be attributed to an aging Brazilian population or, as recent studies hypothesize, may be due to various factors like improved nutrition, medicare, and education [15, 33]. However, the current results suggest that given the data collected, there is still inconclusive evidence on how the age distribution of a municipality relates to risk of VL infection. As mentioned previously, due to the nature of the data observed, this study relates risk of disease at a municipality level. Therefore, results cannot explain variability of age effects on VL risk at an individual level.

The present study revealed spatially heterogeneous VL risks and demonstrates opportunities for mesoregion and municipality-specific public health policy interventions. Fig 9 shows most municipalities in the southwest West Potiguar mesoreigon have average relative risk estimates ranging between two and six times the expected intensity if there was no spatial or temporal variability in disease outcomes in RN. Between 2007 and 2020, Fig 10 shows municipalities in West Potiguar consistently had higher VL relative risk estimates than municipalities in the Central, Agreste and Eastern mesoregions. Specifically, we see that the southwest municipalities are highly likely to have risk of VL more than double the expectation. West Potiguar borders the states of Ceará and Paraíba, who have also experienced increasing incidence rates of VL over time, respectively [6, 8]. The exceedance probabilities of relative risk greater than two for the Central and Agreste Potiguar regions range between 0% and 80% across time, with a few northern municipalities showing high exceedance probabilities during the earlier years of this study. Whereas the majority of the municipalities in the Eastern Potiguar region exhibited low exceedance probabilities of the RR being greater than two, suggesting the relative risk of VL in this region is likely lower than 2. S3 Fig, further suggests the municipalities in red are high likely to have a RR greater than one.

The top three municipalities whose estimates exhibited an upward trend include Pau Dos Ferros, Patu, and Natal. Specifically, Natal’s trend cyclically increased until 2016 before trending downward. The cyclical nature of Natal’s estimated risk trend may suggest that another peak could occur in the near future. Such finding of VL behavior in Natal agrees with recent research [15, 33], which characterizes Natal as a city that may require public attention and health policy interventions. Cyclical changes in risk estimates were not unique to Natal as other municipalities’ risk trends shared this cyclical characteristic. These changes in risk may stem from cyclical changes in the environment, such as those brought on from natural phenomena like El Niño/La Niña, which may influence sandfly density through climate alterations [12, 53]. Future research is needed to explore the variables that are creating this upward cyclic trend in VL risk. Also observed are Mossoro’s and Açu’s downward risk trends during the study period. Mossoro and Natal are the two most populous municipalities in RN. Further research is warranted to understand their diverging risk trends.

Another noteworthy phenomenon the present model identified is in the coastal metropolis regions of East Potiguar. Natal, Nisia Floresta, and Extremoz displayed high RR of VL within this mesoregion, but despite it’s geographical proximity to these municipalities, Parnamirim’s risk trend was estimated to be lower. Parnamirim’s lower risk indicates the number of VL cases observed in this municipality were lower than what we expected based on the overall prevalence of disease in RN and the municipality’s population over time. Future research to identify the differing epidemiological drivers of VL around Natal is warranted.

### Limitations

Visceral leishmaniasis is a complex disease. We do not claim the present set of covariates embodies an exhaustive representation of the epidemiology of visceral leishmaniasis or risk of the disease in RN, or that we have included all of the most relevant covariates that influence a municipality’s regional disease risk. Climate information, such as precipitation or humidity, may be fruitful to incorporate in future investigations as the RN region is affected by cycles of drought which could interfere with sand fly density [54].

It is noteworthy to mention that we treated the EFA factors as fixed covariates, which assumes the factor scores are known and not estimated. This assumption makes the model partially Bayesian as it did not account for uncertainty when estimating these factors. However, we believe this lack of uncertainty is minimal since the factor scores were estimated irrespective of the outcome of interest (VL cases). Additionally, the covariate risk factors used in the EFA were treated as point-in-time observations since 2010 was the only available Census collection within the study time period, at the time of this writing. Incorporating temporal Census covariates could augment future model inference.

Geographical sparsity of observed VL counts was also a limitation; the data did not contain VL cases for a total of 47 municipalities throughout the 14-year study period. In our study, municipalities without documented VL counts were treated as missing data and our model predicted the outcomes for those regions. However, the true missingness mechanisms are unknown, and the proposed approach may still acquire bias.

The location used for each disease case in this study was also a limitation. Depending on the data available, the location of each VL case was either the municipality of infection or the municipality of diagnosis. First, we note that the municipality of diagnosis might not be the municipality of residence for all patients since some individuals might have traveled to larger cities due to limited healthcare services in small/rural cities. Due to this limitation in the observed data, the results in this study might underreport disease risk in rural regions. Secondly, while the municipality of infection can help identify disease reservoirs and therefore inform on the population at risk, municipality of infection was not observed for every participant in the study. When municipality of infection was not observed then the municipality of diagnosis was used. We believe that individuals in the municipality of diagnosis are also at risk since they are exposed to infected individuals that travel to these areas for diagnosis purposes. Therefore, we do not expect that using either locations in the analysis would affect the estimated parameters, inference, or risk maps results since we believe the population at risk can include the populations in either municipality.

Finally, the present study contained positive VL cases which were medically confirmed under passive surveillance. However, VL prevalence relies on disease transmission rates that are interdependent of animal reservoirs, sandfly reservoirs, and human reservoirs, which were not observed for the present analysis. Integrating these data sources in future research is important to better inform more localized and individual risk of disease.

## Conclusion

The purpose of this manuscript was to introduce a modeling approach to evaluate disease risk while addressing shortcomings in data collected such as missing information and correlated covariates. The results focus on estimated risk of visceral leishmaniasis across Rio Grande do Norte, Brazil between 2007 and 2020 after adjusting for potential risk factors at a municipality level. We applied a hierarchical Bayesian approach to estimate municipality-specific relative risk of VL across space and time. Our results show that poor socioeconomic status and lower livestock production are related to increases in VL risk in RN. Estimates indicate it is highly likely that many municipalities within the West Potiguar mesoregion of RN have VL risk larger than double what would be expected if there was no spatio-temporal disease heterogeneity. The present observations revealed spatially heterogeneous VL risks and demonstrated opportunities for municipality-specific public health policy interventions. Additionally, our model estimated that the risk of VL in Natal, and possibly in Patu and Pau dos Ferros, is increasing over time which warrants future research to identifying epidemiological drivers in those areas.

## Supporting information

S1 Fig Spearman’s correlation coefficients between 2010 Brazilian Federal Census variables. (TIF)

S2 Fig Spearman’s correlation coefficients between three Social Vulnerability Indices that were constructed using the 2010 Brazilian Federal Census variables. (TIF)

S3 Fig The spatial and temporal posterior probability that a municipality’s relative risk estimate shown in Fig 9 is greater than one. Darker shades of red indicate it is likely the municipality’s observed VL cases were greater than that number expected based on overall prevalence of VL in RN and the municipality’s population size. This figure was created using the geobr package in R [25].
(TIF)

S4 Fig The trends plotted how municipality-specific posterior mode estimates of RR over time within the four mesoregions in RN. The red represents a relative risk of on.
(TIF)

S1 Table Factor loadings for the three factors estimated in the Exploratory Factor Analysis. (PDF)
