# Supplementary figures and images for: Hierarchical spatiotemporal modeling of human visceral leishmaniasis in Rio Grande do Norte, Brazil
Source: PLoS Negl Trop Dis. 2023 Apr 3;17(4):e0011206. doi: 10.1371/journal.pntd.0011206 (PMC10101641; doi:10.1371/journal.pntd.0011206)

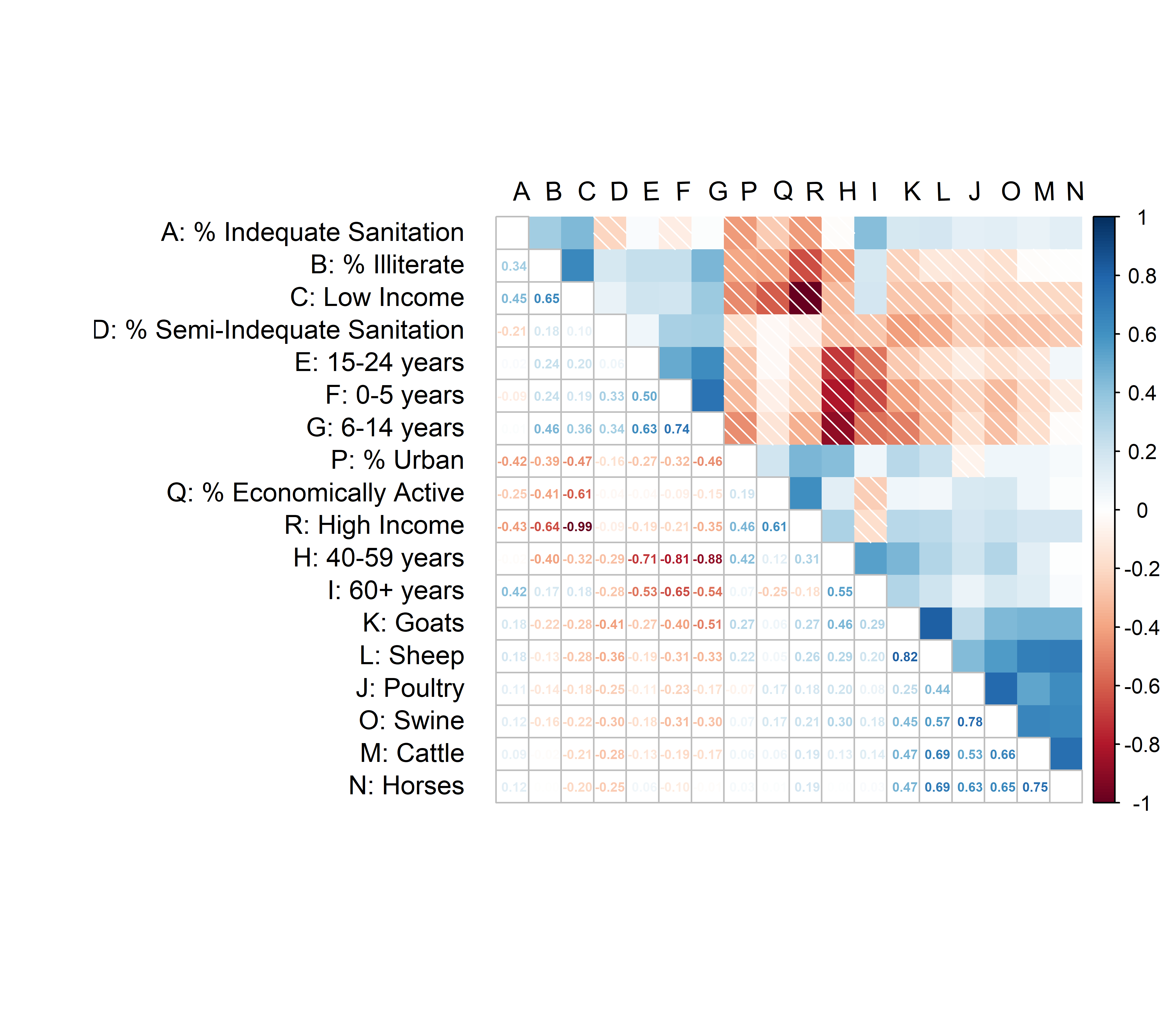

Supplement: S1 Fig — (TIF) [file pntd.0011206.s001.tif]

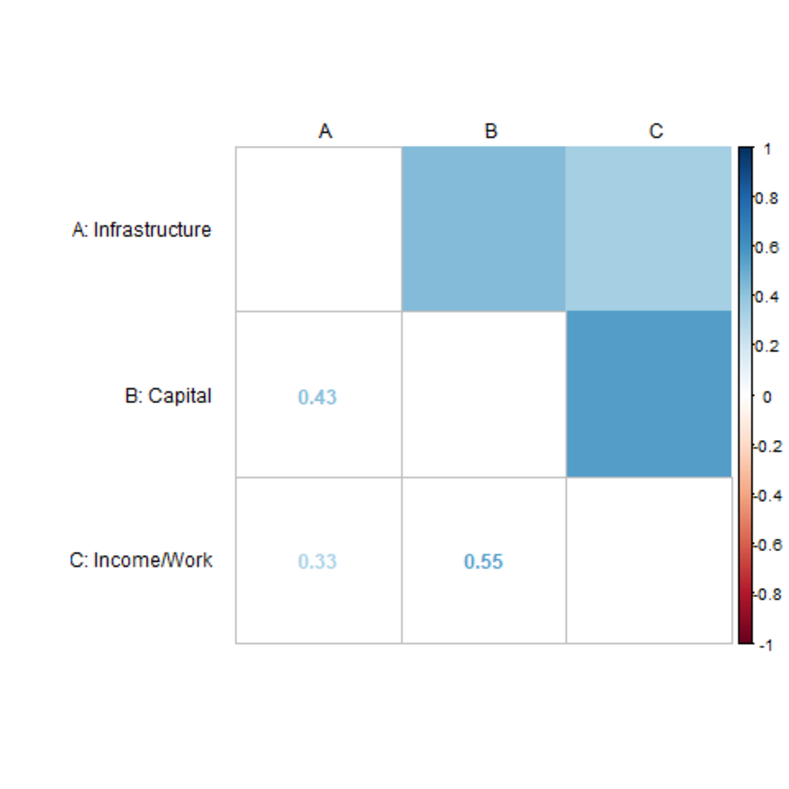

Supplement: S2 Fig — (TIF) [file pntd.0011206.s002.tif]

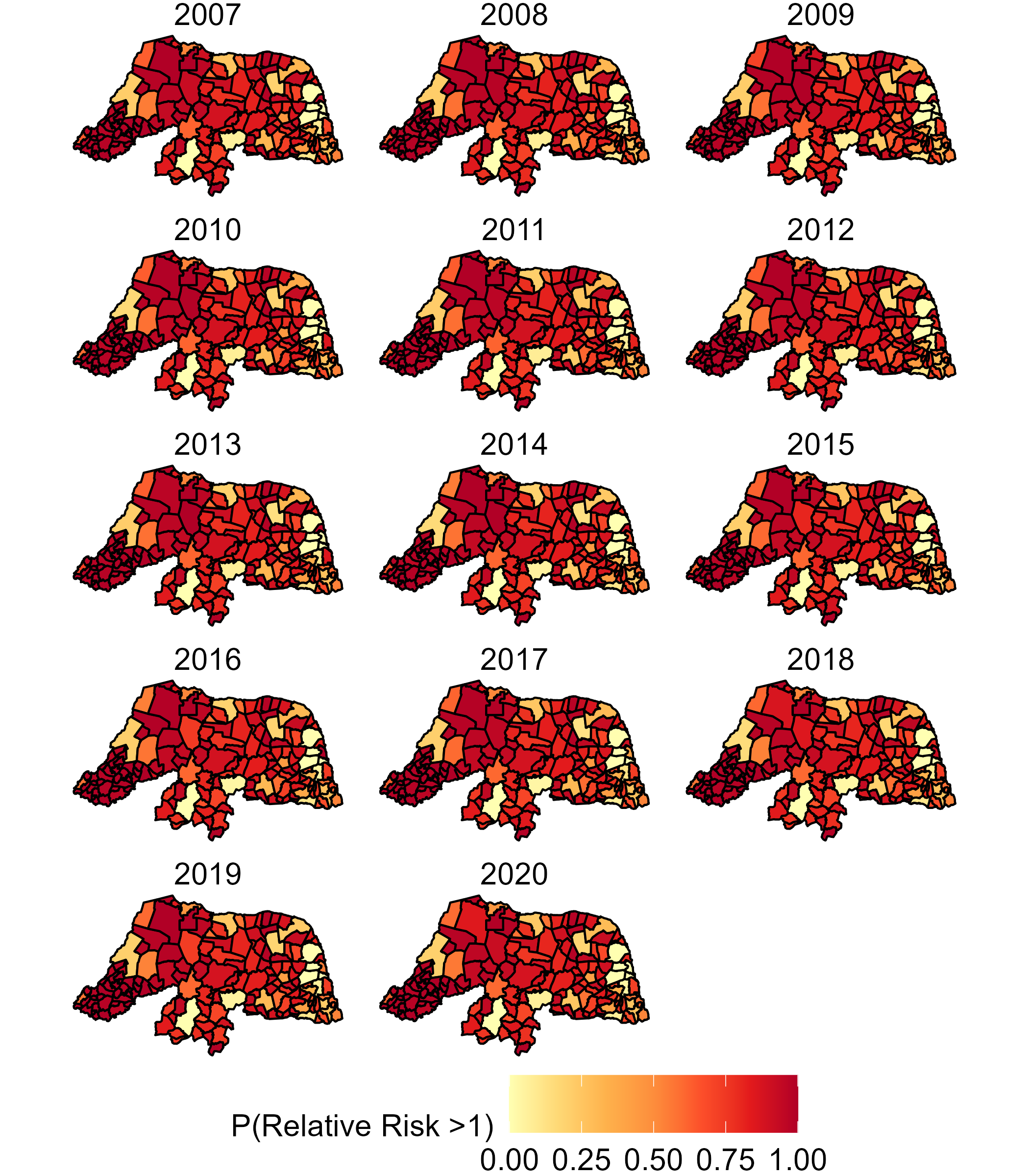

Supplement: S3 Fig — The spatial and temporal posterior probability that a municipality’s relative risk estimate shown in Fig 9 is greater than one. Darker shades of red indicate it is likely the municipality’s observed VL cases were greater than that number expected based on overall prevalence of VL in RN and the municipality’s population size. This figure was created using the geobr package in R [25]. (TIF) [file pntd.0011206.s003.tif]

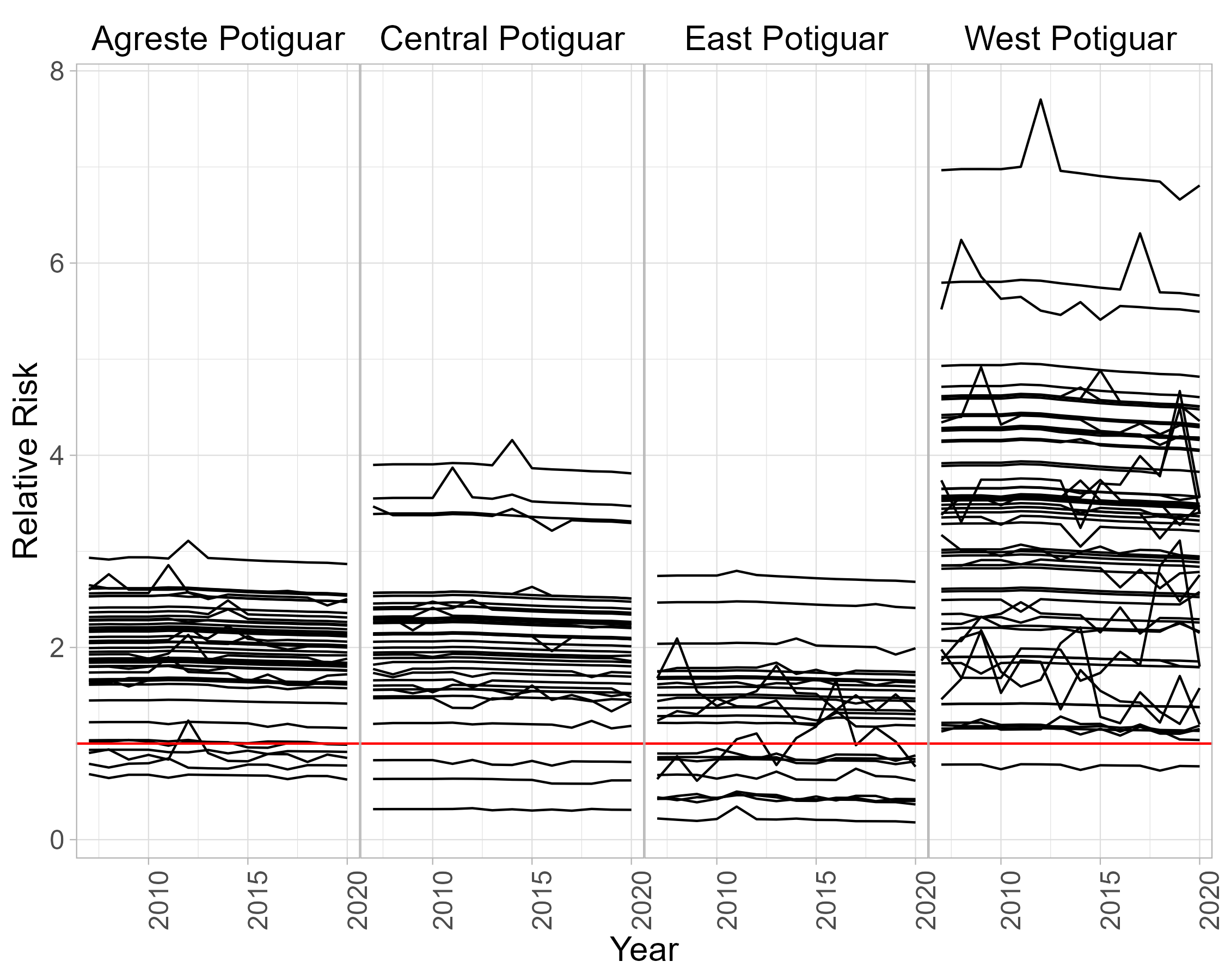

Supplement: S4 Fig — The trends plotted how municipality-specific posterior mode estimates of RR over time within the four mesoregions in RN. The red represents a relative risk of on. (TIF) [file pntd.0011206.s004.tif]
