# Supplementary material for: Hierarchical spatiotemporal modeling of human visceral leishmaniasis in Rio Grande do Norte, Brazil
Source: PLoS Negl Trop Dis. 2023 Apr 3;17(4):e0011206. doi: 10.1371/journal.pntd.0011206 (PMC10101641; doi:10.1371/journal.pntd.0011206)
Supplement: S1 Table — (PDF) [file pntd.0011206.s005.pdf]

Table S.1. Factor loadings for the three factors estimated in the Exploratory Factor Analysis.

Census Covariate	Socioeconomic Status	Age Distribution	Livestock Production
Percent Urban	-0.47	-0.34	
0 – 5 years		0.82	-0.19
6 – 14 years	0.27	0.84	-0.18
15 – 24 years		0.68	
40 – 59 years	-0.24	-0.92	0.13
60+ years	0.46	-0.77	
Percent Illiterate	0.75	0.24	-0.15
Percent Semi-Inadequate Sanitation		0.33	-0.29
Percent Inadequate Sanitation	0.55		
Percent Economically Active	-0.55		0.11
Cattle Production			0.79
Goats Production		-0.22	0.69
Horses Production	-0.15		0.86
Poultry Production	-0.40		0.34
Sheep Production		-0.22	0.90
Swine Production	-0.20	-0.17	0.80
Low Income	0.95	0.15	-0.17
High Income	-0.94	-0.15	0.15
